# Supplementary material for: Cutaneous lupus features specialized stromal niches and altered retroelement expression
Source: J Invest Dermatol. Author manuscript; Available in PMC 2025 Aug 16. (PMC12354343; doi:10.1016/j.jid.2025.04.033)
Supplement: Supplementary Material (PIIS0022202X25004889) [file NIHMS2087912-supplement-Supplementary_Material__PIIS0022202X25004889_.pdf]

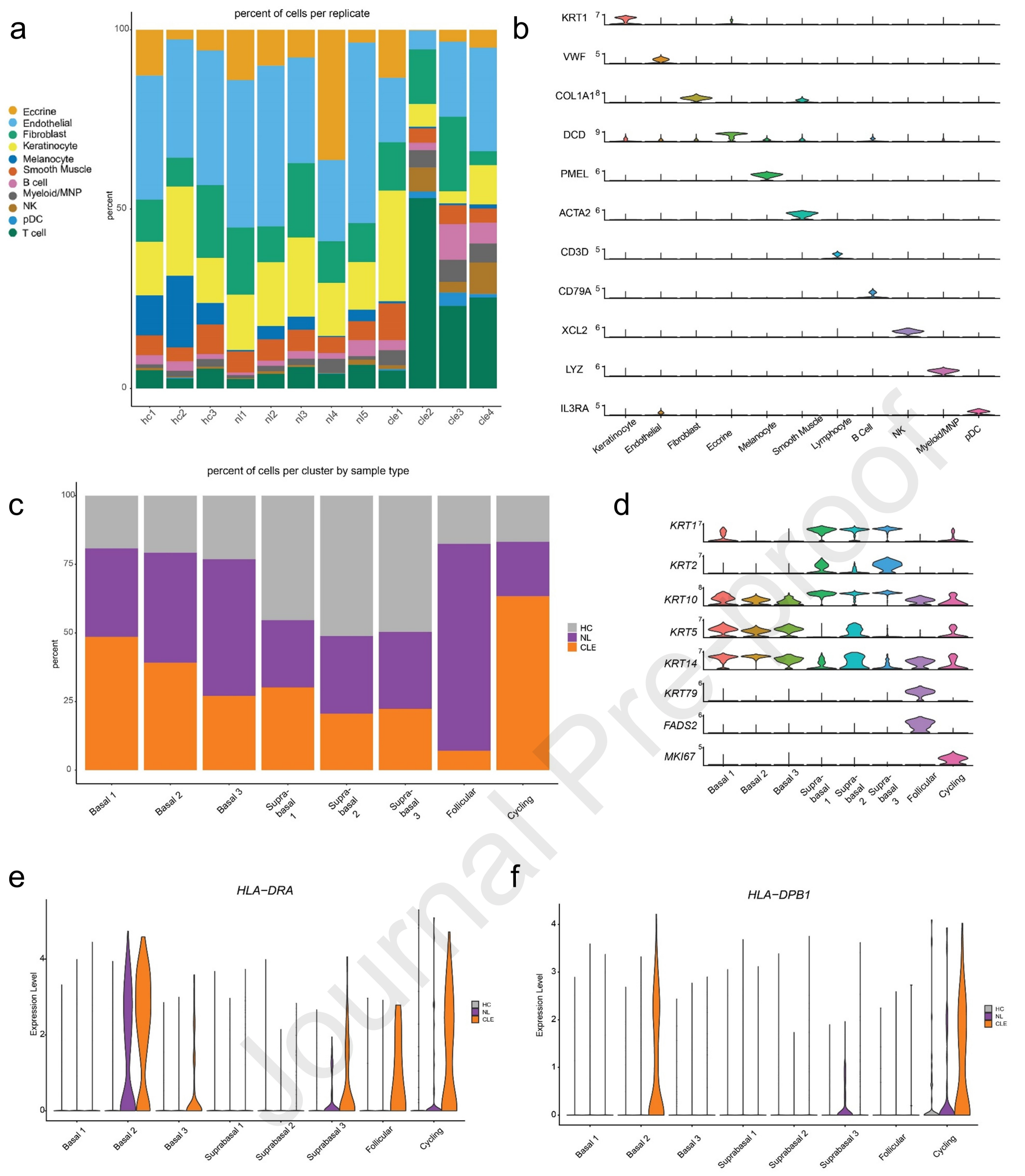


Journal Pre-proof

Journal Pre-proof

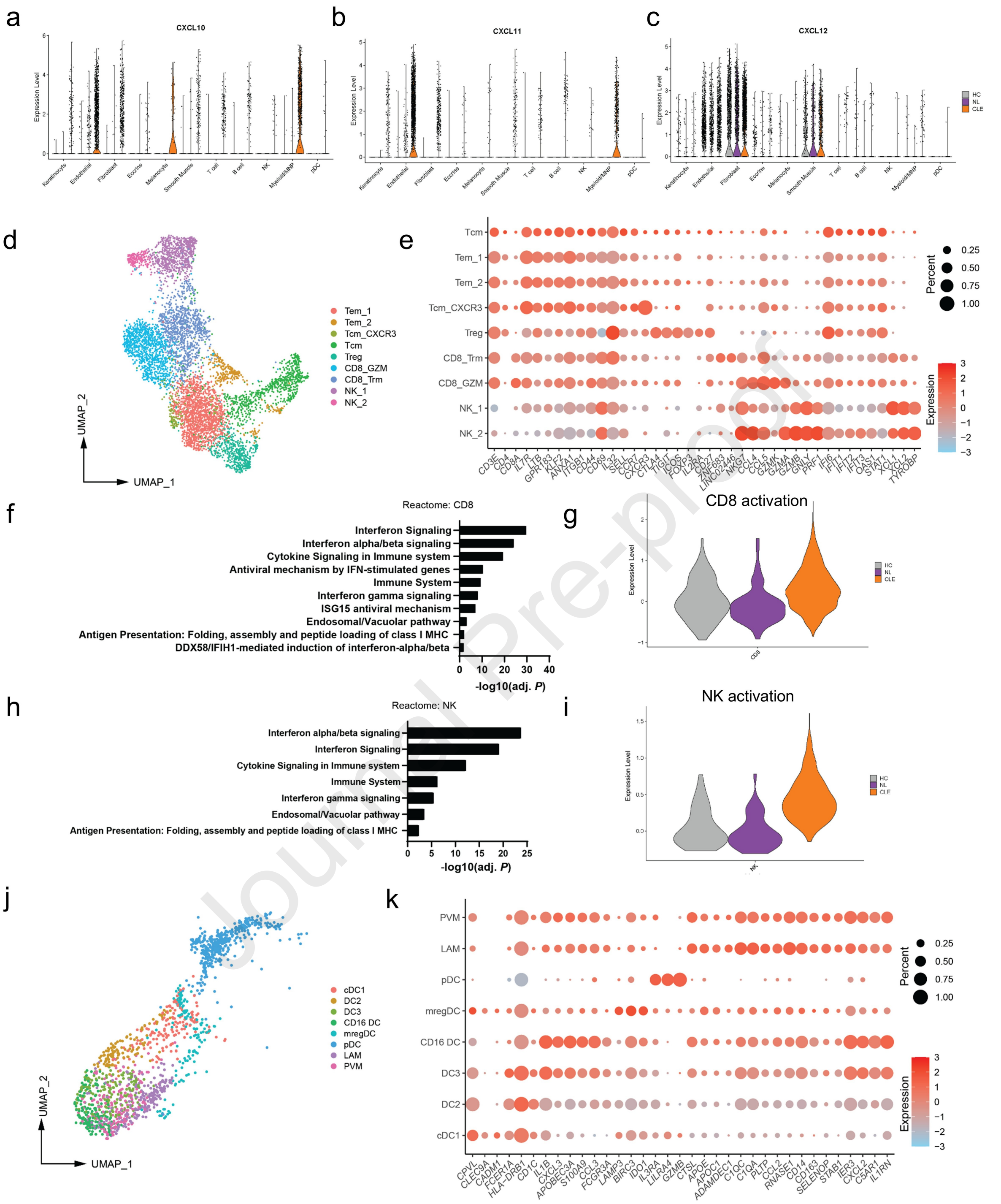

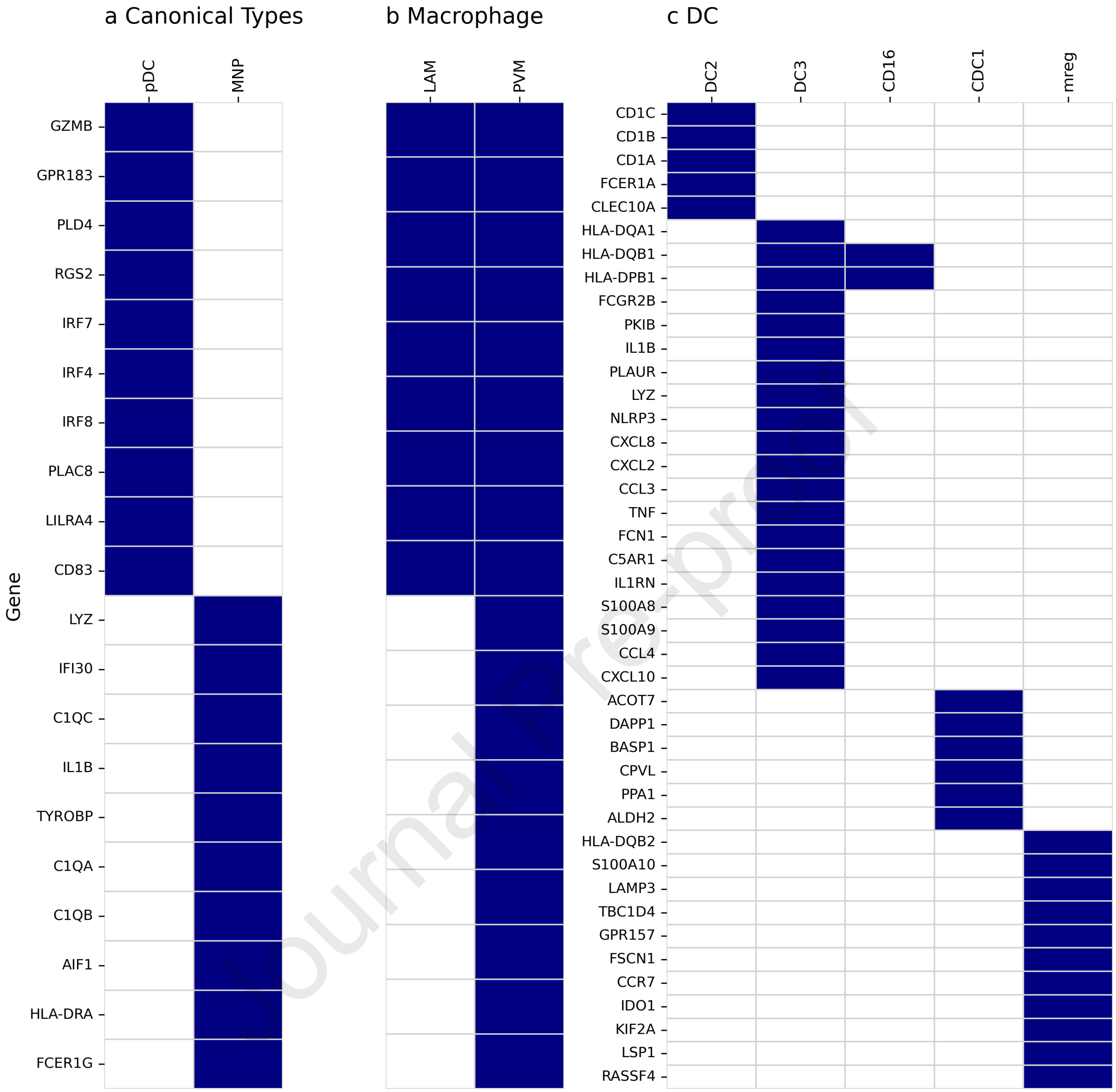
a Canonical Types
b Macrophage
c DC
pDC
MNP
LAM
PVM
DC2
DC3
CD16
CDC1
mreg
Gene
GZMB
GPR183
PLD4
RGS2
IRF7
IRF4
IRF8
PLAC8
LILRA4
CD83
LYZ
IFI30
C1QC
IL1B
TYROBP
C1QA
C1QB
AIF1
HLA-DRA
FCER1G
CD1C
CD1B
CD1A
FCER1A
CLEC10A
HLA-DQA1
HLA-DQB1
HLA-DPB1
FCGR2B
PKIB
IL1B
PLAUR
LYZ
NLRP3
CXCL8
CXCL2
CCL3
TNF
FCN1
C5AR1
IL1RN
S100A8
S100A9
CCL4
CXCL10
ACOT7
DAPP1
BASP1
CPVL
PPA1
ALDH2
HLA-DQB2
S100A10
LAMP3
TBC1D4
GPR157
FSCN1
CCR7
IDO1
KIF2A
LSP1
RASSF4

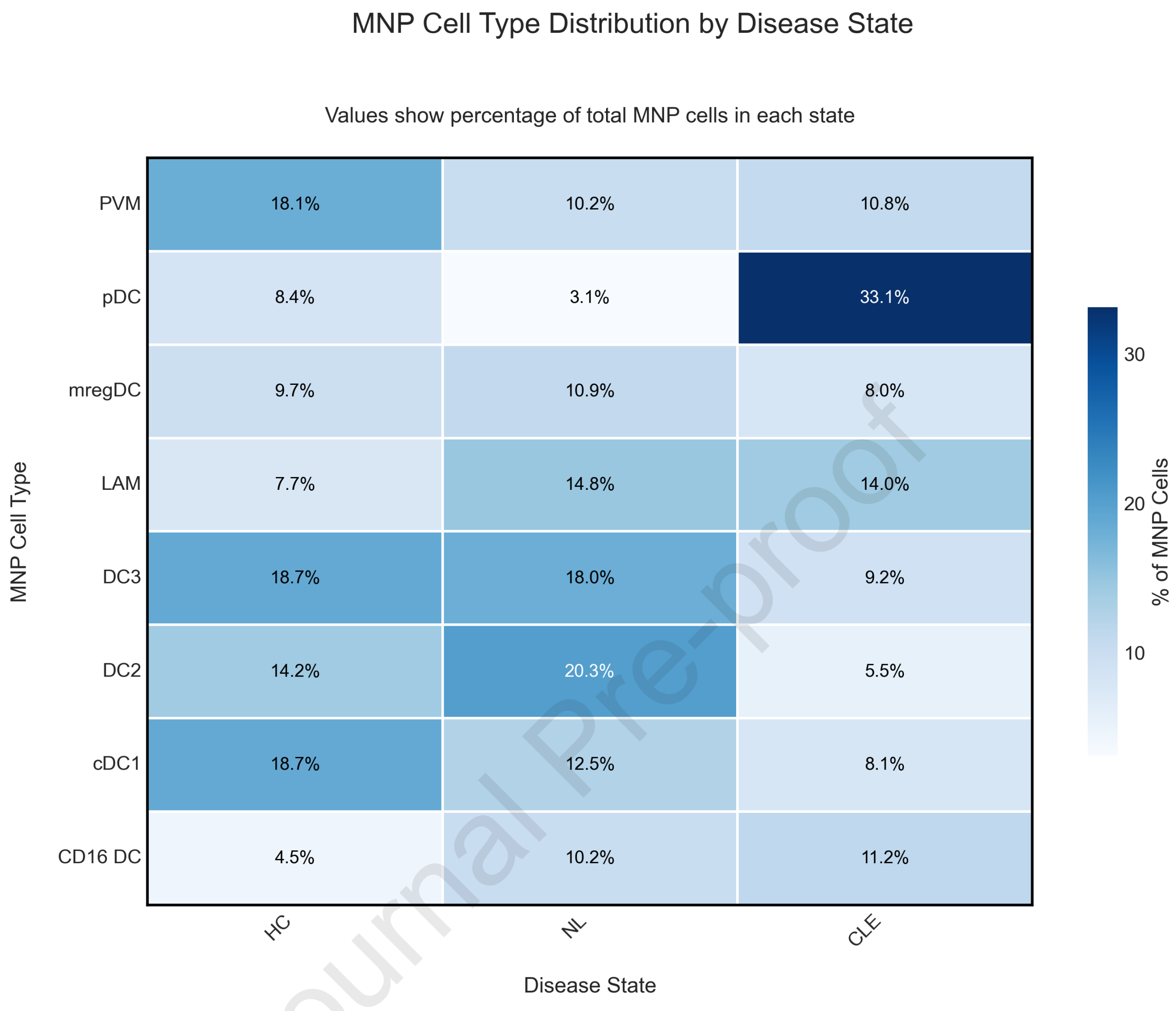


Journal Pre-proof

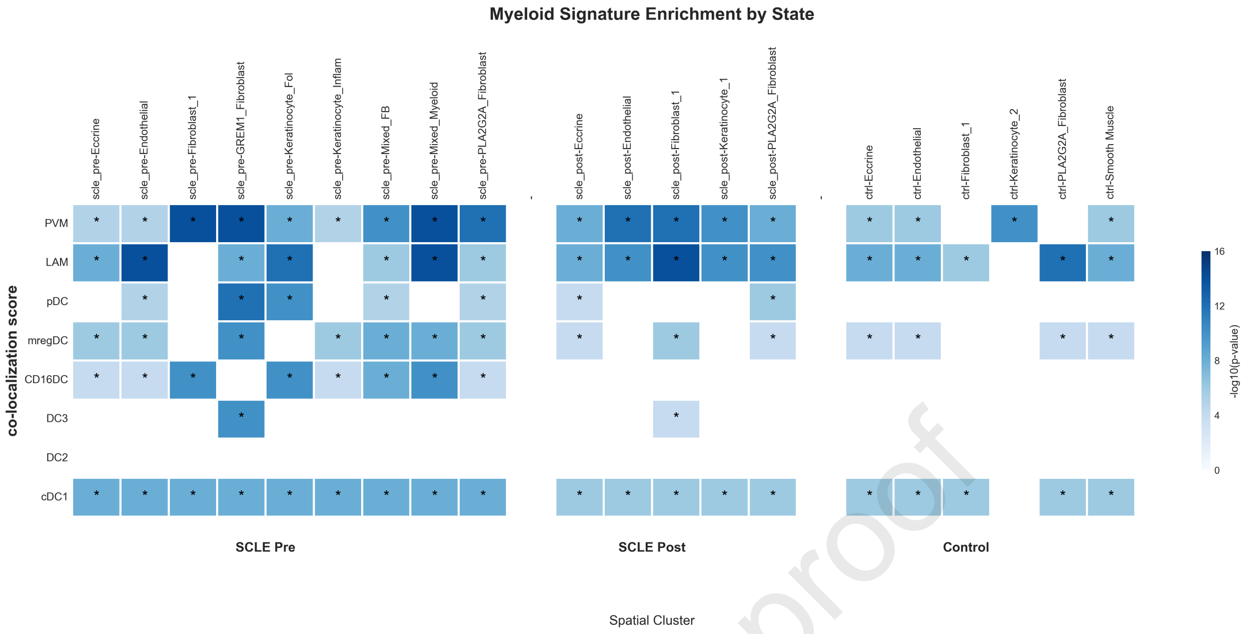


Journal Pre-proof

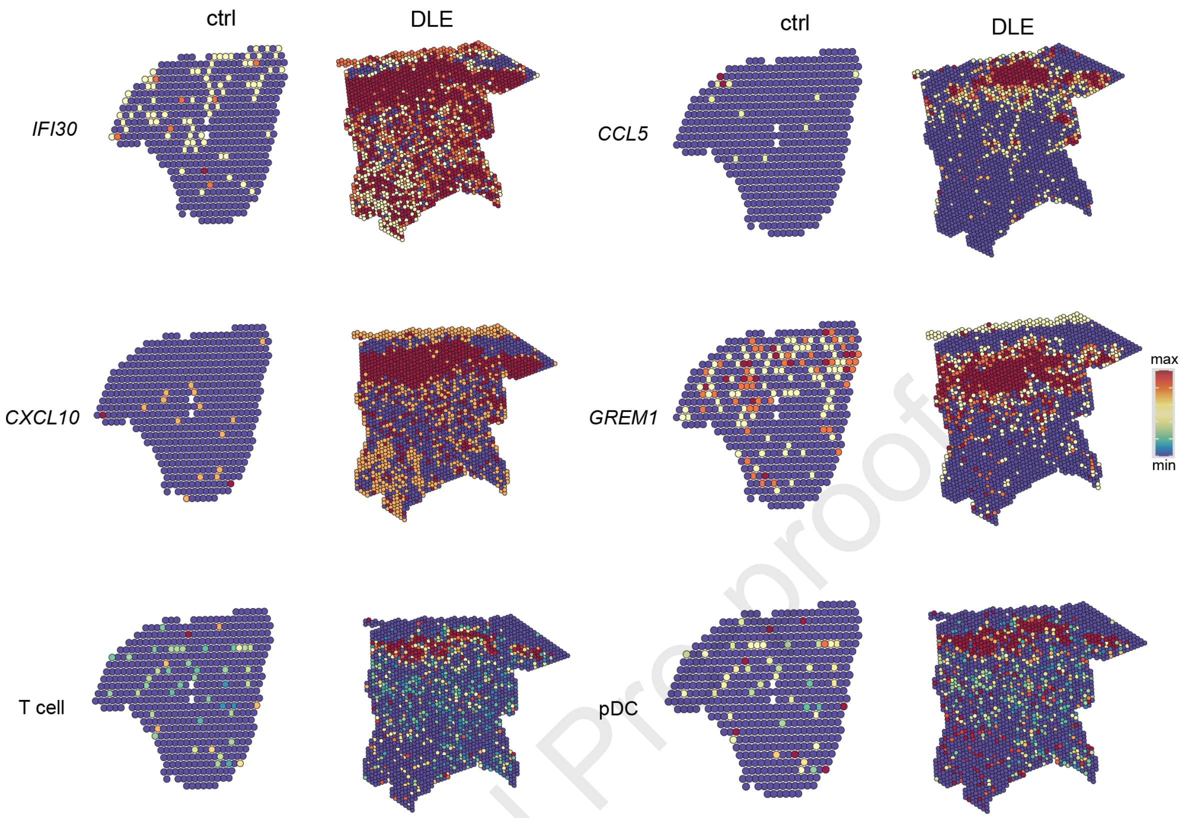


Journal Pre-proof

Journal Pre-proof

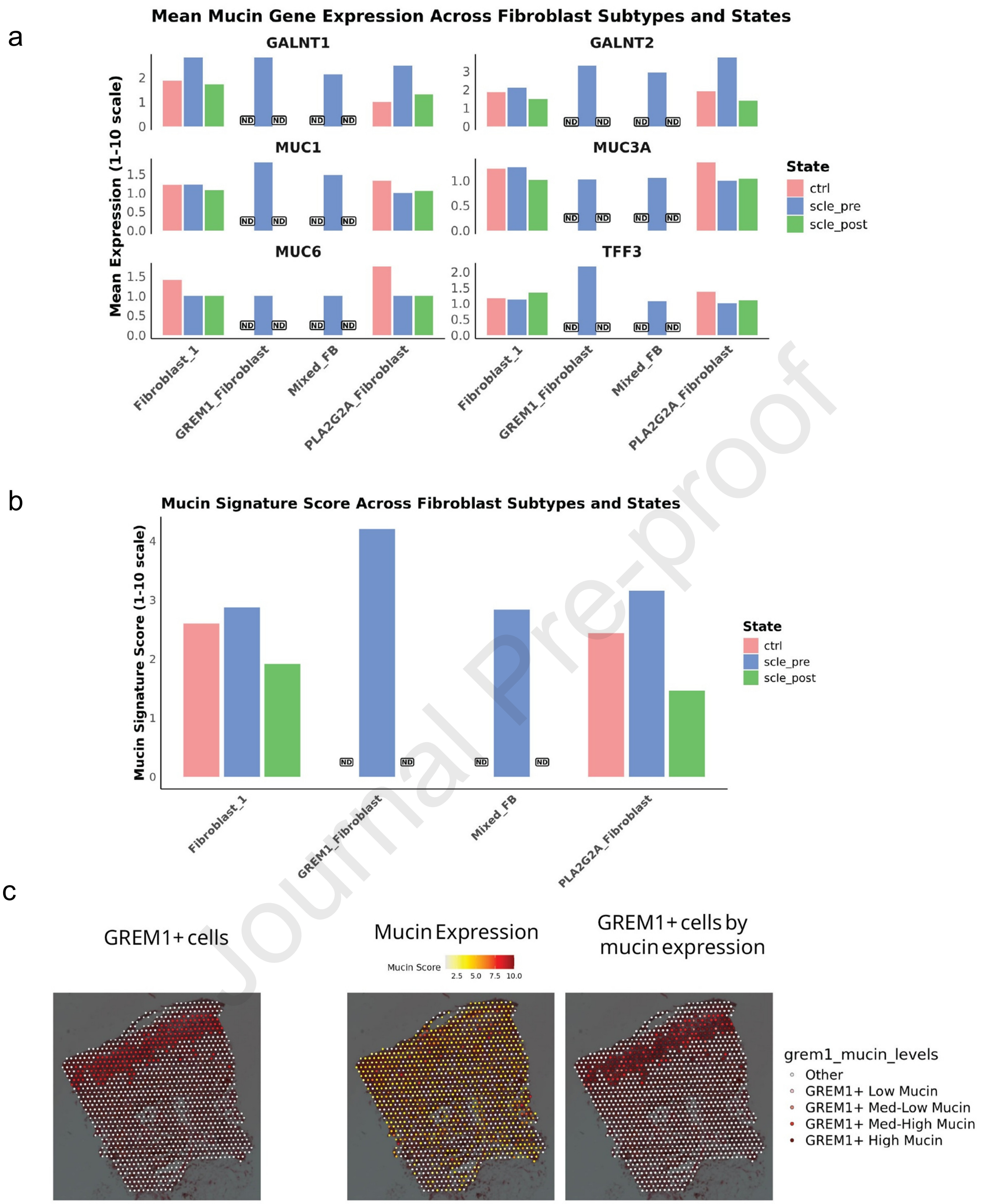


Journal Pre-proof

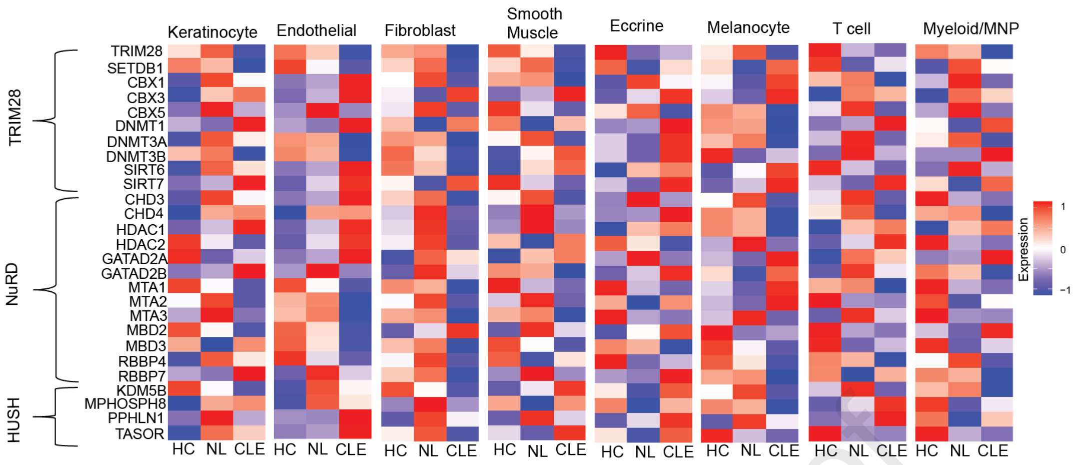


Journal Pre-proof

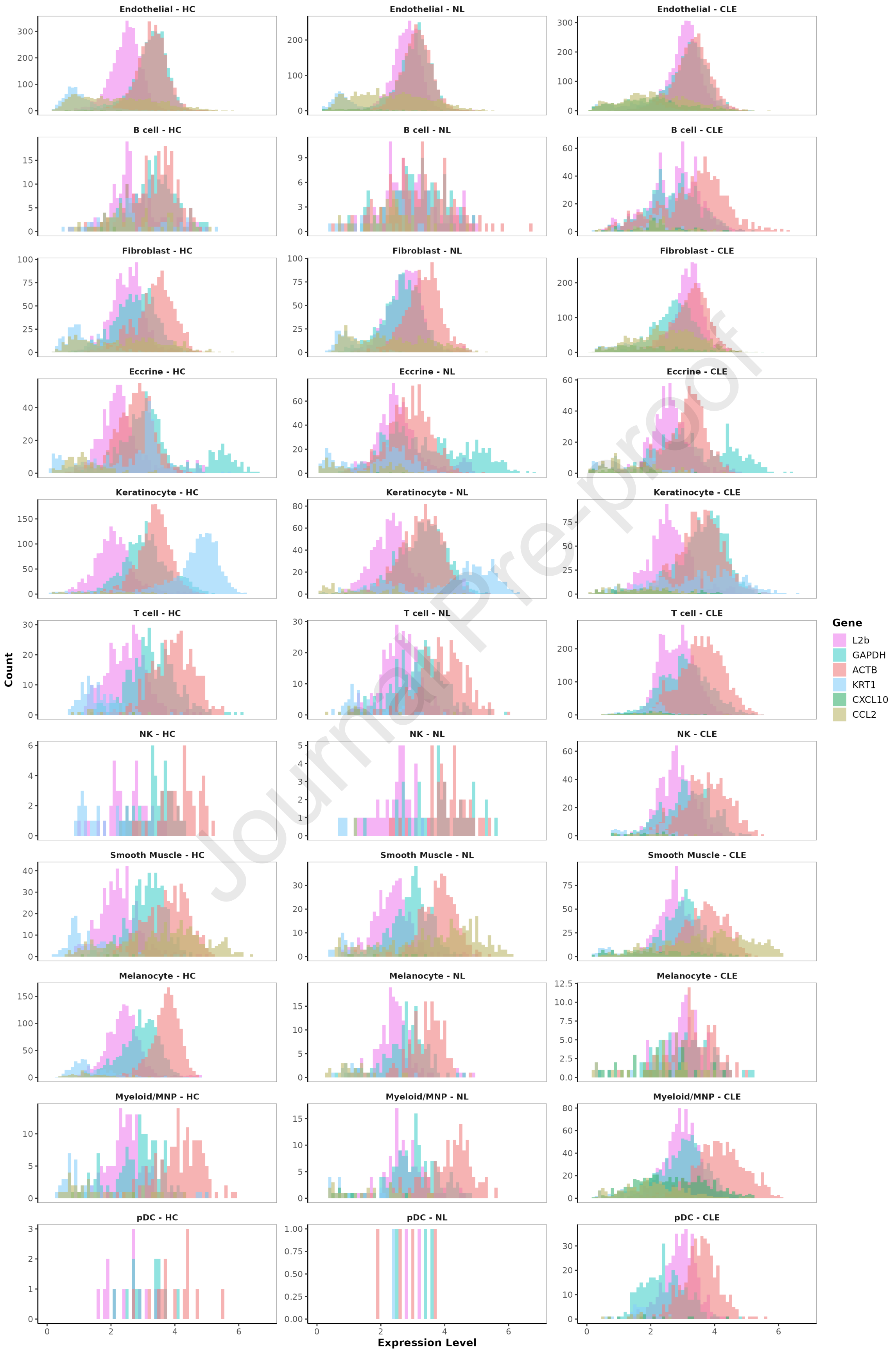


Journal Pre-proof

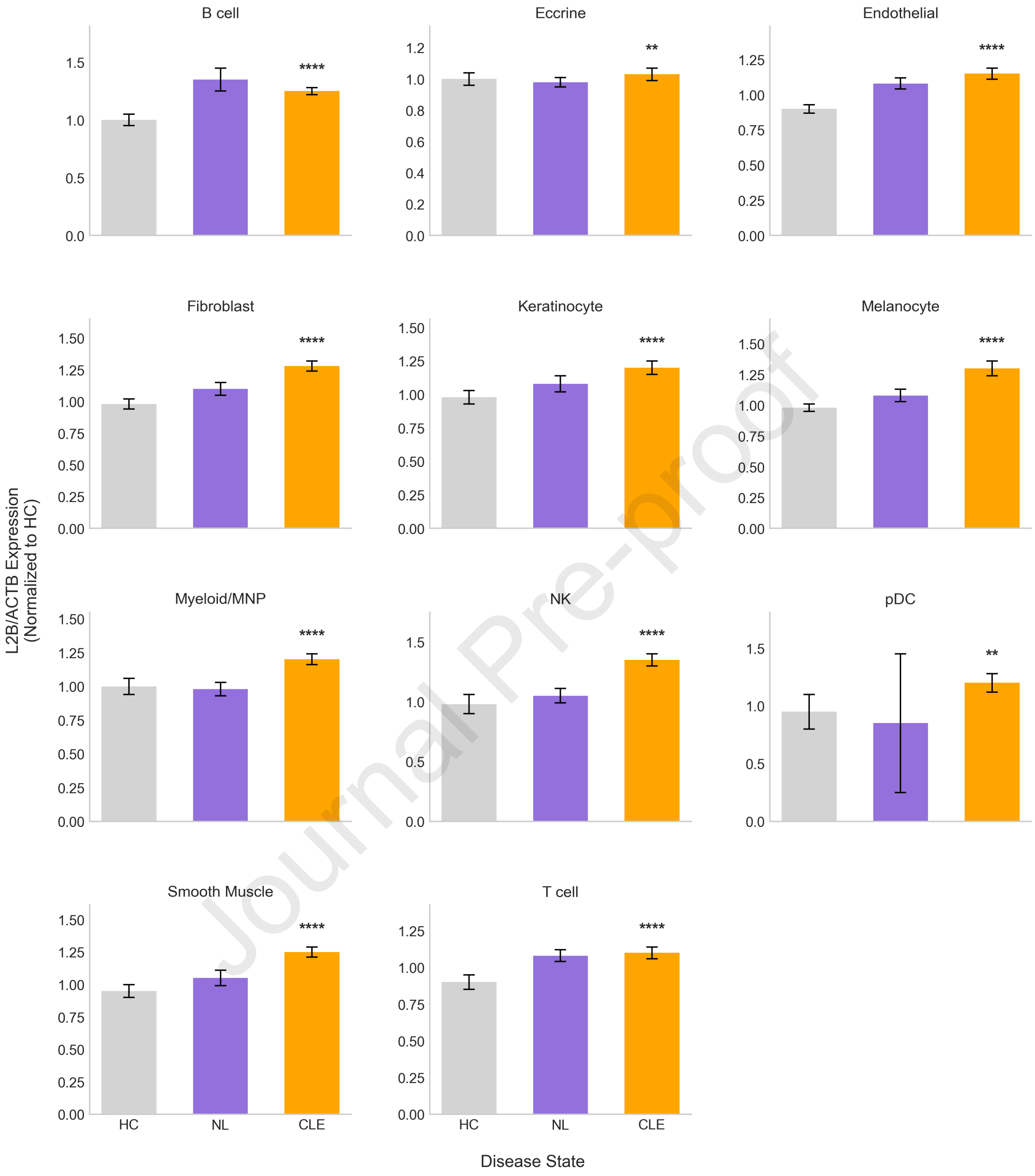


Journal Pre-proof

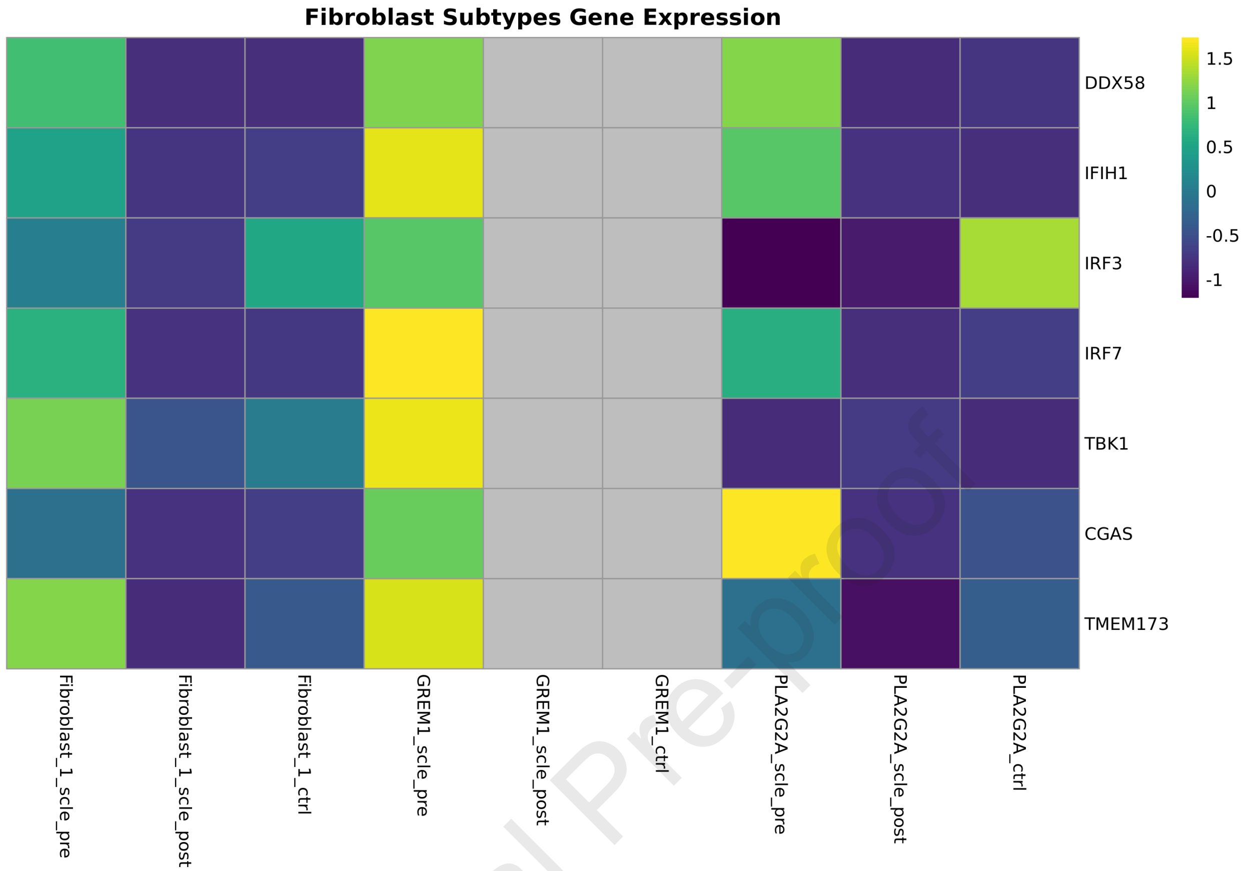


Journal Pre-proof

Journal Pre-proof

| Assay | Patient group | Sex | Age | Skin Type | CLE type | Site | SLE/CLE medications |
|---|---|---|---|---|---|---|---|
| Single cell | CLE (+ SLE) | F | 27 | 5 | DLE | Arm | HCQ 200 mg BID + Belimumab 200 mg/ml weekly |
| Single cell | CLE | F | 26 | 2 | DLE | Arm | HCQ 200 mg BID |
| Single cell | CLE | F | 43 | 5 | DLE | Face | None |
| Single cell | CLE | F | 47 | 5 | DLE | N/A | HCQ 200 mg BID + MMF 2500 daily |
| Single cell | CLE | F | 42 | 5 | DLE | Back | Topical Clobetasol 0.05% ointment, MTX 15 mg weekly, ILK |
| Single cell | HC | F | 29 | 3 | N/A | Back | N/A |
| Single cell | HC | M | 40 | 2 | N/A | Back | N/A |
| Single cell | HC | F | 34 | 5 | N/A | Back | N/A |
| Spatial | CLE (+SLE) | F | 43 | 3 | SCLE | Arm | Pre: HCQ 200 BID + Prednisone 5 daily + Lenalidomide 5 mg qod |
| Spatial | [redacted] | | | | | | Post: HCQ 200 BID + Prednisone 5 daily + Anifrolumab 300 mg q4w |
| Spatial | CLE | F | 53 | 5 | DLE | Thigh | None |
| Spatial | HC | F | 39 | 2 | N/A | Arm | N/A |

**Supplementary Table 1: Patient and Sample Characteristics.** Table of patients enrolled in study including basic demographics and disease-specific medications. BID = twice daily, DLE = discoid lupus erythematosus, SCLE = subacute cutaneous lupus, HCQ = hydroxychloroquine, ILK = intralesional kenalog, MMF = mycophenolate mofetil, MTX = methotrexate, qod = every other day.

Supplementary Figure legends

**Supplementary Figure 1: Cell Type Distribution and Marker Expression Profiles in CLE and Healthy Skin Samples.** A) Bar plot showing distribution of cell types by sample. B) Violin plot showing unique marker gene expression segregated by cell types. C) Bar plot showing distribution of cells per cluster, annotated by tissue state. D) Stacked violin plot showing unique marker gene expression of different keratinocyte populations. F and G) Violin plots of MHC II genes showing unique elevations in CLE keratinocytes.

**Supplementary Figure 2: Lymphoid and Myeloid Cell Characterization in CLE.** A, B and C) Violin plots of indicated chemokines annotated by cell type and tissue cell state. D) UMAP of Lymphoid cell subclustering. E) Bubble plot of lymphoid cell marker gene expression displayed by cell type. Color indicates relative expression levels between cell types in dataset and the size indicates the percentage of cells per cluster expressing the particular gene. F and H) Reactome pathway analysis of upregulated genes in CD8 T cells (F) and NK cells (H) of CLE cells compared with HC. G and I) Violin plot of CD8 T cell cytotoxicity score and NK cell activation scores (see methods). J) UMAP plot of MNP cell subclustering. K) Bubble plot of MNP cell marker gene expression displayed by cell type.

**Supplementary Figure 3: Markers for Myeloid/MNP Cell Subpopulations in Skin.** Top 10 cell marker genes for each subclustered MNP cell type. Subclustered cell types include: cDC1, DC2,

Journal Pre-proof

DC3, CD16+ DC, mregDC, LAM (lipid associated macrophage), and PVM (perivascular macrophage).

**Supplementary Figure 4: Altered MNP Cell Type Distribution Across Disease States.** MNP subclustered cell types, separated by tissue state. Subclustered cell types include: cDC1, DC2, DC3, CD16+ DC, mregDC, LAM (lipid associated macrophage), and PVM (perivascular macrophage).

**Supplementary Figure 5: Differential Spatial Co-localization of Myeloid Cells in Pre/Post Treatment Conditions.** Cell type co-localization analysis through multimodal intersection analysis, highlighting differences of Myeloid/MNP populations in different tissue states. Subclustered cell types include: cDC1, DC2, DC3, CD16+ DC, mregDC, LAM (lipid associated macrophage), and PVM (perivascular macrophage).

**Supplementary Figure 6: Spatial Distribution of Key Inflammatory Genes and Cell Signatures in DLE versus Healthy Control Skin.** Spatial gene expression plots of the indicated genes or cell type signatures (T cell, pDC) in HC (left) and DLE (right).

**Supplementary Figure 7. Mucin Gene Expression in CLE Fibroblast Subtypes Shows Treatment-Responsive Changes to Anifrolumab.** A) Examination of mucin gene expression in different fibroblast clusters in pre, post-treatment, and control states. ND = cell type was not present in tissue state. ctrl = healthy control skin; scle_pre = subacute cutaneous lupus skin biopsy prior to treatment; scle_post = subacute cutaneous lupus skin biopsy post treatment with Anifrolumab (paired samples from same patient, biopsied 8 weeks a part). B) Aggregate mucin gene expression (*GALNT1, GALNT2, MUC1, MUC3A, MUC6, TFF3*) in different fibroblast clusters in pre, post-treatment, and control states. ND = no cell types were present in that tissue state. C) Aggregate mucin gene expression (*GALNT1, GALNT2, MUC1, MUC3A, MUC6, TFF3*) overlaid on *GREM1*+ fibroblasts populations in CLE. The left panel plots *GREM1*+ fibroblasts and the middle panel plots the aggregate mucin gene expression. In the right panel, GREM1+ fibroblasts are plotted on the basis of low, medium, and high expression of mucin. ctrl = healthy control skin; scle_pre = subacute cutaneous lupus skin biopsy prior to treatment; scle_post = subacute cutaneous lupus skin biopsy post treatment with Anifrolumab (paired samples from same patient, biopsied 8 weeks a part).

**Supplementary Figure 8: Epigenetic Regulator Expression Patterns Vary by Cell Type and Disease State.** Heatmap comparing expression of different epigenetic regulators segregated by tissue state and cell type.

**Supplementary Figure 9: L2b Retroelement Expression Compared to Housekeeping Gene Distribution.** Histogram of cell counts (y axis) and expression levels (x axis) of selected genes, including housekeeping genes (ACTB, GAPDH) as well as retroelement L2b.

**Supplementary Figure 10: Elevated L2b Retroelement Expression Across Multiple Cell Types in CLE Compared to Healthy Controls.** L2b levels normalized to HC levels for all cell types and tissue states. Wilcoxon rank sum test comparing CLE and HC, * = $P < .05$, ** = $P < .01$, *** = $P < .001$, **** = $P < .0001$.

**Supplementary Figure 11: Treatment with Anifrolumab Modulates RLR and cGAS-STING Pathway Expression in Fibroblast Subpopulations.** Heatmap of RLR and cGAS-STING pathway gene expression pre and post-Anifrolumab treatment in Fibroblast clusters. These are fibroblast clusters identified in **Figure 4B**. Gray columns indicate that the fibroblast cell type is not present in that tissue state.

Journal Pre-proof
